# Bilateral Simultaneous Revision Total Knee Arthroplasty as a Single Staged Procedure: A Case Report and Review of Literature

**DOI:** 10.7759/cureus.1112

**Published:** 2017-03-23

**Authors:** Raju Vaishya, Amit Kumar Agarwal, Chirag Jaiswal, Vipul Vijay, Abhishek Vaish

**Affiliations:** 1 Orthopaedics, Indraprastha Apollo Hospitals; 2 Orthopaedics, Indraprastha Apollo Hospital

**Keywords:** revision, total knee arthroplasty, bilateral, wear, loosening

## Abstract

Bilateral revision total knee arthroplasty (TKA) is a surgical procedure, which is rarely done simultaneously as it is a difficult surgery and the safety of simultaneous bilateral single stage surgery remains unknown. We report a case of a 67-year-old woman who presented to us with bilateral painful and unstable TKA (right > left) of six months duration. The primary bilateral TKA were done 14 years ago. Bilateral simultaneous revision TKA was performed, using cemented, constrained, long-stem prostheses. The intraoperative and postoperative periods remained uneventful. At last follow-up at four years, she had a pain-free range of motion of up to 0–115°, and the patient had returned to the activities of daily living. She had stable knees with good function and no evidence of loosening or wear.

## Introduction

Total knee arthroplasty (TKA) is a promising treatment for end-stage osteoarthritis (OA) of the knee for alleviating pain and restoring the function of the knee. Some of the cases with bilateral TKA are symptomatic, necessitating revision arthroplasty in both the knees. A bilateral revision TKA can be done either in two stage or simultaneously as a single stage procedure. However, the decision to perform simultaneous bilateral revision TKA is debatable because of possible higher complexity and complication rate. Very few cases have been reported in the literature on this issue. There are various advantages of doing simultaneous bilateral revision TKA compared with staged bilateral revision TKA. These include single operation and single anesthesia as well as better rehabilitation of both knees, apart from a significant reduction in the hospital stay and hospital costs.

## Case presentation

A 67-year-old hypothyroid and hypertensive female presented to us with unstable and painful knees 14 years after primary bilateral TKA for advanced OA. She began developing pain in both the knees for last six months, followed by instability in both knees (right > left). She was managed symptomatically with painkillers, bracing, and physiotherapy but her pain and instability were not relieved.

**Figure 1:**
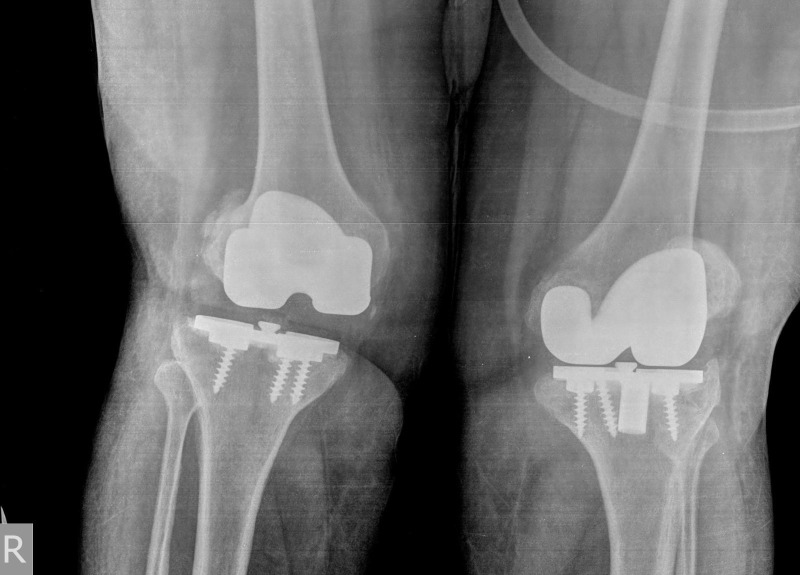
Preoperative anteroposterior (AP) standing radiograph showing bilateral failed total knee arthroplasties (TKAs).

**Figure 2:**
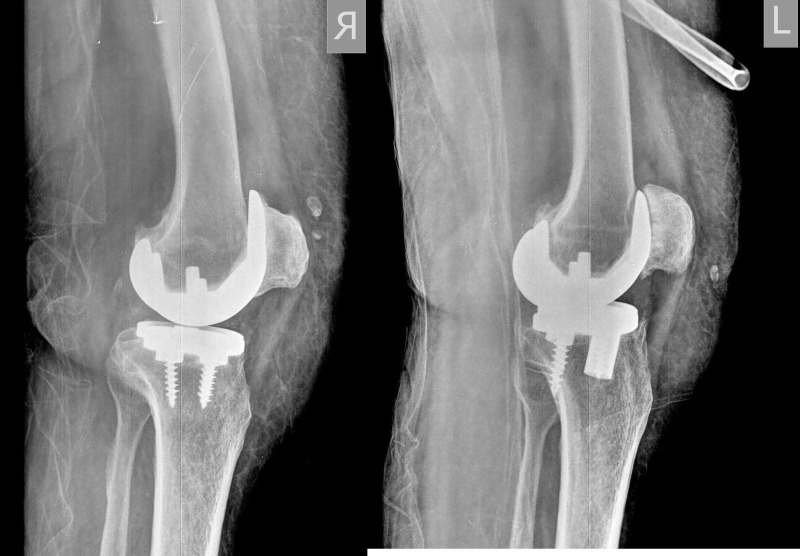
Preoperative lateral radiographs of both knees showing bilateral failed total knee arthroplasties.

On clinical examination, the active and passive knee range of motion was painful. The flexion was 0° to 100°, anterior–posterior laxity of 5–10 mm, and a mild valgus laxity. The plain radiographs showed malalignment and loosening of the implants (Figures 1-2). The leucocyte counts, C-reactive protein, and erythrocyte sedimentation rate (ESR) were within normal limits. A three-phase bone scan was also found to be negative for infection.

**Figure 3:**
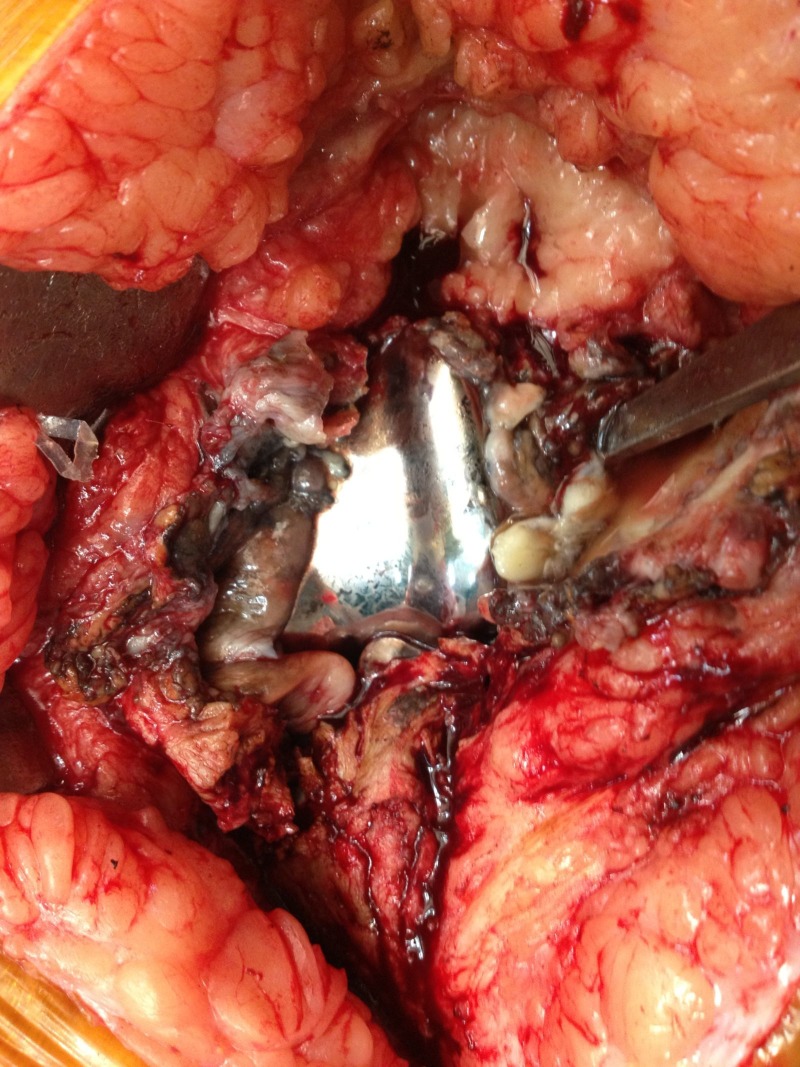
Intraoperative picture showing implants from the right knee with extensive debris and significant wear of the polyethylene insert.

Bilateral revision TKAs were performed using modified Insall’s midline approach with lateral retraction of the patella (Figure 3) [1]. A joint wound swab was taken and sent for gram stain, culture, and sensitivity. It was found to be negative for any microorganisms. The original cemented TKA implants were removed carefully, preserving as much bone as possible. Revision TKA was done on both sides sequentially, under the same anesthesia, using Scorpio® Total Stabilizer (Stryker®, Mahwah, NJ) constrained implants with long femoral and tibial stems.

**Figure 4:**
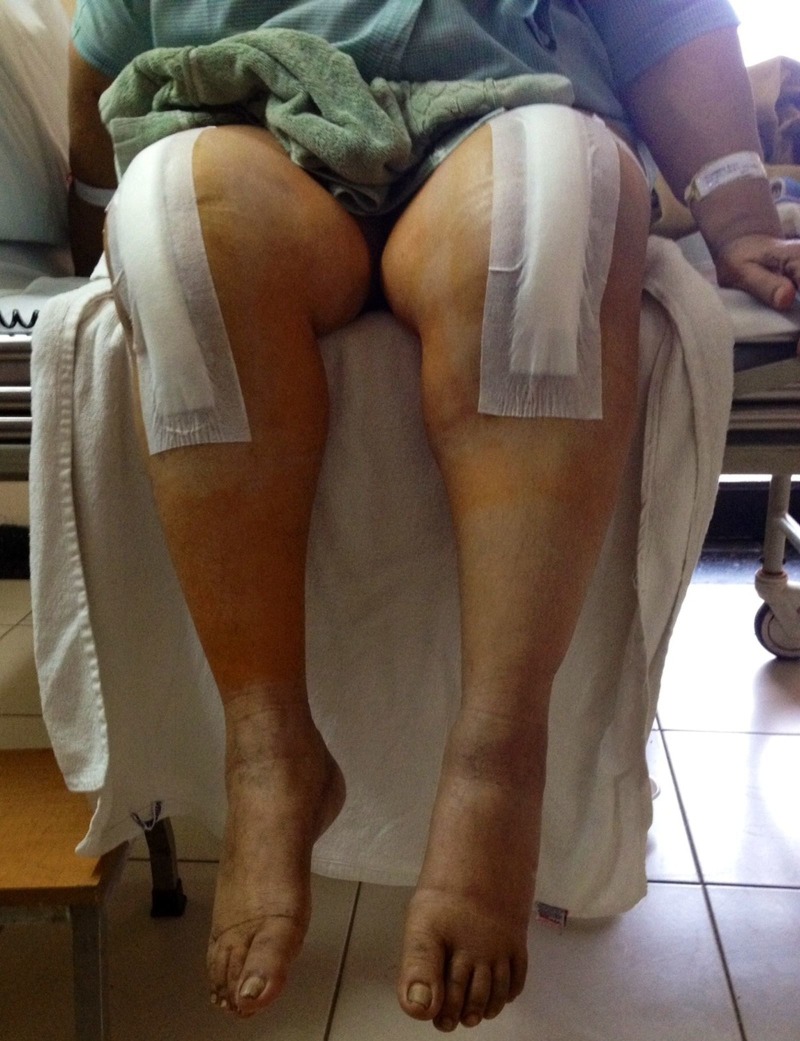
Pain-free range of knee motion (0-90 degrees) after bilateral revision total knee arthroplasties in immediate postoperative period.

The knees were protected in hinged braces postoperatively. The drains were removed 48 hours postoperatively; continuous passive motion (CPM) and active knee flexion exercises were started on postoperative day one and gradually increased to 0°–90° of flexion (Figure 4).

**Figure 5:**
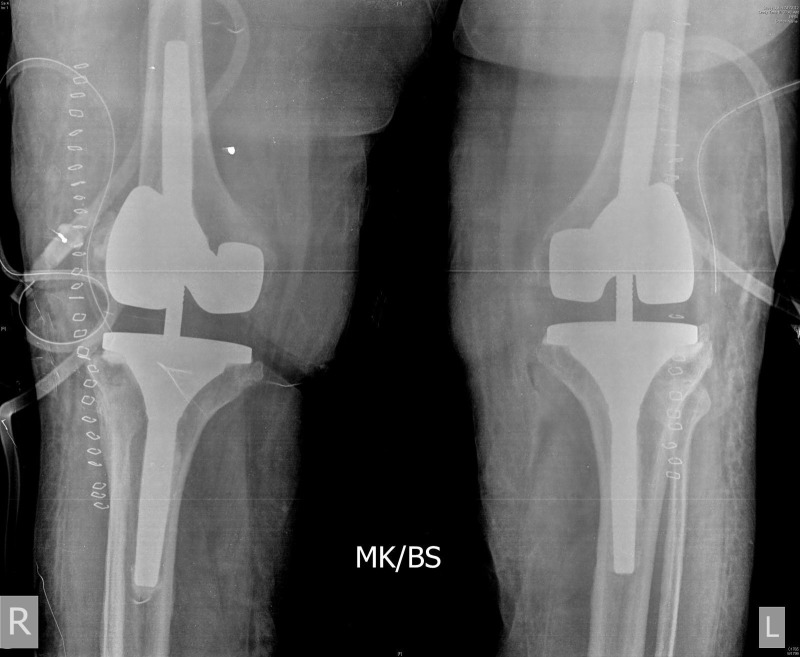
Postoperative AP radiographs after bilateral revision total knee arthroplasties showing well aligned new constrained implants in both knees.

**Figure 6:**
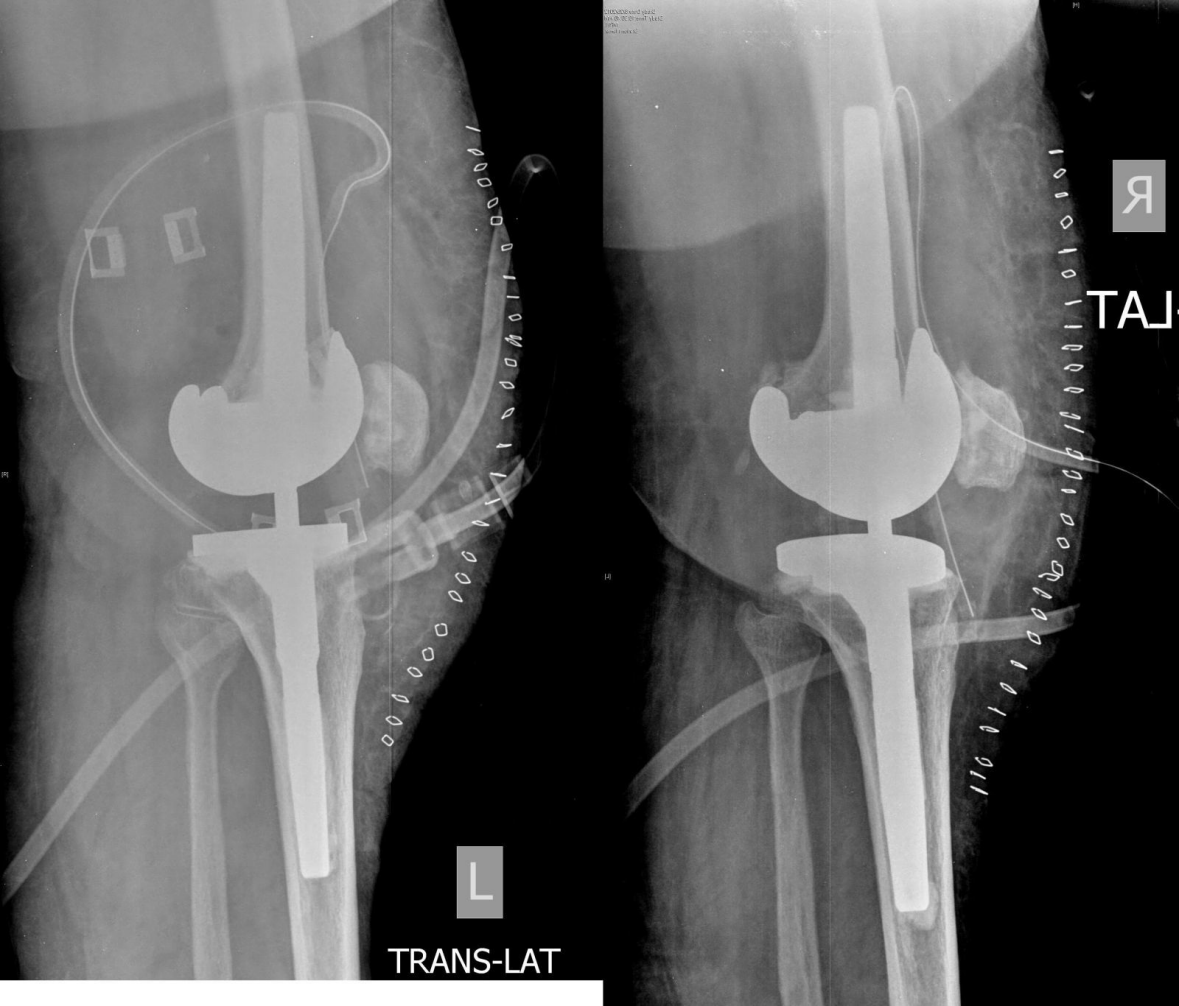
Postoperative lateral radiographs showing well-aligned new constrained implants in both the knees.

The postoperative radiographs showed satisfactory implant positions (Figures 5-6). The patient had no complaints and was able to flex the knee to 80° easily. The range of motion and quadriceps strengthening exercises continued without forced flexion. She gradually resumed full weight-bearing with the help of the walker. Three months after surgery, the brace was removed, and active pain-free range of motion of 0°–115° was achieved with complete stability. At four months, the patient had returned to full activity without the brace or cane. At the final follow-up of four years, the knee was fully stable, and the patient was pain-free with no loosening or wear of the implants.

## Discussion

Symptomatic instability and pain following primary TKA requires revision surgery. In one retrospective study of 49 TKA patients with bilateral simultaneous revision, no postoperative cardiovascular complications, stroke, or death were noted [2]. The minor reported complications included transient, self-limited confusion (in three cases); pulmonary embolism (in one patient), which was treated successfully with an inferior vena cava filter and extended anticoagulation; posterior compartment syndrome (in one case), which was treated by fasciotomy; and stiff knee in one patient (that was manipulated under anesthesia at three months). In a retrospective cohort study, Carter, et al. [3] found that 33 of 141 morbidly obese patients (23.4%) who had revision TKA had a complication compared to 10 of 96 patients with a BMI 18.5 - 25 (10.4%) (p = 0.011). The most common complication was wound healing.

Kevin, et al. reviewed 60,355 revision TKA procedures done in the USA and noted that the most common causes of revision TKA were an infection in 25.2%, implant loosening in 16.1%, and implant failure/breakage in 9.7% cases [4]. They found that revision of all the components was the most common type of procedure done (35.2%). Singh, et al. found a high prevalence (46.5%) of overall moderate to severe activity limitation at two years and 50.5% at five years following revision TKA [5]. Significantly higher odds of moderate to severe overall activity limitation was noted both at two and five-year follow-ups in patients with a BMI of 40 or higher, age greater than 80 years, higher Deyo-Charlson score, and in females.

Kasmire, et al. studied predictors of functional outcome after revision TKA by using various parameters, such as short-form 36 (SF-36), Western Ontario and McMaster Osteoarthritis Index (WOMAC), and Knee Society Scores (KSS) [6]. The data was collected preoperatively and at two years follow-up in their 175 revision TKAs done for aseptic failure. All of the above-mentioned parameters improved significantly after revision TKA (p < 0.001). Lower preoperative pain and higher clinical KSS were found to be predictors of a better outcome.

Sheth, et al. found that the complication rates were different for bilateral TKA done simultaneously and as staged procedures [7]. These authors reported aseptic revision (1.17% vs. 0.9%), septic revision (0.8% vs. 0.7%), mortality (0.28% vs. 0.1%), and adverse events (2.49% vs. 1.97%). According to Bohm, et al., simultaneous bilateral primary TKA patients required more blood transfusions, a shorter hospital stay, more transfers to a rehabilitation facility, and less frequency of knee infections than staged bilateral TKA patients [8]. However, these patients had a higher rate of cardiac complications and in-hospital mortality rate. The three-year revision, however, was same in both the groups.

In a meta-analysis of 14 studies, Hu, et al. showed that the prevalence of mortality immediately postoperatively, mortality at 30 days postoperatively, and neurological complications were significantly higher in simultaneous TKA compared to staged TKA patients [9]. The prevalence of thromboembolic disease, infection, and cardiac complications were not significantly different between simultaneous TKA compared to staged TKA patients. According to Hersekli, et al., the amount of blood loss, intensive care unit days and perioperative complications were same between single- and two-staged operations (p > 0.05) [10]. However, hospital stay and overall cost were significantly less in single-staged operations.

We faced the challenge in decision-making regarding the staging of the procedures in this reported case, where revision of the components was necessary for both knees. We could not find proper guidelines regarding bilateral revision TKA as there are only a few documented reports of simultaneous bilateral revision TKA. There is limited evidence to support the one-stage practice of doing bilateral revision TKAs, as its safety remains controversial. We chose to do a single-staged bilateral revision TKA in this case, as a two-staged procedure would have required two anesthesias, longer hospital stay, more hospital bills, and surgery-related complications, which were overcome by a single-staged procedure in this case. With the use of constrained implants and long stems of the prosthetic components, we achieved good knee stability and satisfactory range of motion immediately postoperatively and at the four year follow-up.

## Conclusions

Two-staged bilateral revision total knee replacement (TKA) has many disadvantages, such as requiring anesthesia to be given twice, a longer hospital stay, more hospital bills, and higher surgery-related complications, which can be overcome by a single stage procedure. In carefully selected patients, single-staged bilateral revision TKAs should be considered over two-staged procedures.
